# Origin of Cave Fungi

**DOI:** 10.3389/fmicb.2018.01407

**Published:** 2018-06-28

**Authors:** Zhi-Feng Zhang, Peng Zhao, Lei Cai

**Affiliations:** ^1^State Key Laboratory of Mycology, Institute of Microbiology, Chinese Academy of Sciences, Beijing, China; ^2^College of Life Science, University of Chinese Academy of Sciences, Beijing, China

**Keywords:** BEAST, geologic age, molecular clock, obligate troglobitic fungi, speciation

## Abstract

Karst caves are obviously characterized by darkness, constantly low temperature, high humidity, and oligotrophy. Previous studies revealed that Karst caves have a high and specific bio-diversity. A large number of troglobiont animals had been discovered and their evolution and speciation have been well investigated. However, the origin and evolution of cave fungi remain unknown. In a previous study, we have identified 20 new species, which accounted for 49% of the total number of new species of fungi ever described from caves. In this study, we inferred the divergence times of these 20 new species and compared to the cave formation geologic age. The fossil-calibrated molecular clock showed that the divergence times of these 20 suspected troglobitic fungi are between late Miocene (7.2 Mya for *Metapochonia variabilis*) and late Jurassic (158 Mya for *Gymnoascus exasperates*). While based on the historical geological movement and the paleoclimate of Guizhou, it has been estimated that the development of caves in this area was later than middle Pliocene (3.5–4 Mya). It is therefore concluded that the new species described from these caves are unlikely troglobitic fungi but travelers from other environments. The geographic history of caves appeared to be too short for fungal speciation.

## Introduction

Caves are strongly zonal environment, with unique characteristics determined by the surrounding rock, subterranean water, and karst morphology (Kuzmina et al., 2012; Gabriel and Northup, 2013). It significantly differs from the land surface environment in the darkness, constantly low temperature, high humidity, and oligotrophy (Gabriel and Northup, 2013). As a relatively closed space, cave environment can be affected by various factors, such as the air currents, chemolithoautotrophy, visitors, and water movement (streams or water seeps; Hose et al., 2000; Barton and Jurado, 2007a; Gabriel and Northup, 2013; Ortiz et al., 2014). All these above factors contribute to the biota in caves (Ogórek et al., 2013; Ortiz et al., 2014) and caves were suspected to encompass a high, specific biodiversity (Culver and Holsinger, 1992; Gabriel and Northup, 2013; Vanderwolf et al., 2013; Jiang et al., 2017).

Cave fauna can be classified into four major categories: troglobiont, eutroglophile, subtroglophile, and trogloxene. Troglobiont is a species or population that bound to a subterranean habitat strictly, in other words, true cave life (Sket, 2008). The earliest description of fungi in caves was published by Humboldt in 1794, as described in Dobat (1967), and the first ecological literature of caves was by Megušar (1914). Most of the previous studies were focused on cave fauna and it has been suspected that there might be 50,000–100,000 species of cave animals on the earth (Culver and Holsinger, 1992). Quite many troglobitic animals had been discovered, such as *Typhlobarbus nudiventris* (Chu and Chen, 1982), *Typhlocaridina semityphlata* (Cai, 1995), *Angulifemur tridigitis* (Zhang, 1995), and several species of *Macrochlamys* and *Kaliella* (Li, 2003). A number of studies on the evolution of cave fauna had been published, and several hypotheses for the evolution and origin of cave animals have been proposed, such as adaptive shift hypothesis (Rouch and Danielopol, 1987; Desutter-Grandcolas and Grandcolas, 1996) and climatic relict hypothesis (Barr and Holsinger, 1985; Peck and Finston, 1993).

Microbes play important roles in cave (Barton and Northup, 2007b; Gabriel and Northup, 2013). More than 1150 fungal species in 550 genera have been discovered in caves and mines worldwide by 2017 (Vanderwolf et al., 2013; Zhang et al., 2017) and some suspected obligate troglobitic fungi have been reported, such as *Acaulium caviariforme*, *Aspergillus baeticus*, and *Aspergillus thesauricus* (Vanderwolf et al., 2013). Recently, Zhang et al. (2017) described 20 new species from caves, accounting for 49% new fungal species ever described from caves. However, the origin of cave microbiology was unknown and there is no hypothesis on their evolution and origin. The answer to the question “whether if there is real obligate troglobitic fungus” is important for understanding the origin and evolution of cave fungi. The factors affecting the materials and energy in caves, such as water movement, air currents, and visitors, can also act as pathways of microorganism exchange between subterrane and surface environment (Gabriel and Northup, 2013). Meanwhile, most of the currently discovered fungi from caves were known from other environments (Vanderwolf et al., 2013; Zhang et al., 2017). Zhang et al. (2017) suggested that cave fungi may have an origin from outside environment, as all the recorded genera and most of the identified species are known from other environments.

Molecular clock hypothesis is useful in estimating divergence times (Boutin and Coineau, 2000; Bromham and Penny, 2003). To reveal the origin of fungi in caves, a comparison was made between the divergence times of suspected obligate troglobitic fungi with the independent estimation of cave formation geologic age (Juan and Emerson, 2010). In the present study, six nucleotide sequences of the 20 suspected obligate troglobitic fungi described by Zhang et al. (2017), and some aligned representative species in the phylum Ascomycota were combined to infer a fossil-calibrated phylogeny and estimate divergence times for fungi in caves.

## Materials and Methods

### Cave Information

Suiyang county, located in Guizhou, China, has a typical subtropical monsoon climate. The annual mean temperature is 13.5°C, and the annual rainfall is 1116–1350 mm (Jiang et al., 2012).

**FIGURE 1:**
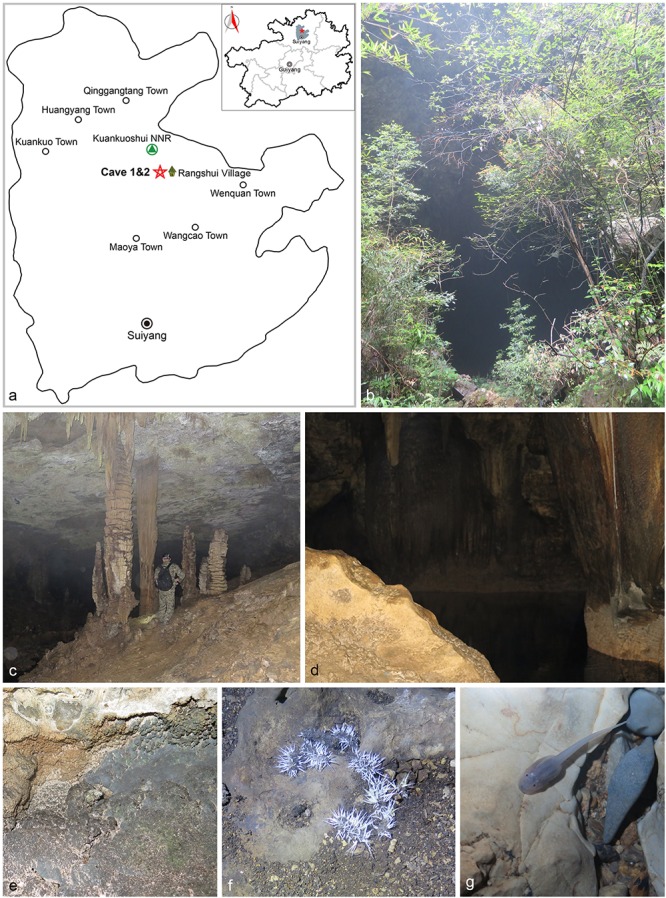
Visited caves. **(a)** Location of caves in Suiyang, Guizhou. **(b)** Entrance to Cave 1. **(c)** Stalactite. **(d)** Pool at the end of Cave 1. **(e)** Sampled rocks. **(f)** Bat guano colonized by fungal mycelia. **(g)** Pellucid tadpole (Zhang et al., 2017).

Two unnamed Karst caves in Wangcao town, Suiyang county, herein named Cave 1 (28° 12′ 629″ N, 107° 13′ 639″ E) and Cave 2 (28° 12′ 599″ N, 107° 13′ 661″ E), are located at the southern edge of Kuankuoshui National Natural Reserve. Both caves are horizontal and zonal and have one entrance hiding in the forest of the hillside (**Figure 1**, Zhang et al., 2017). These two caves are 500 m away from each other, and might belong to the same cave system with subterranean river connection. The elevation of Cave 1 is 908 m; the length is 400 m; the humidity is 75–80%; and the temperature is 21–22°C. The elevation of Cave 2 is 930 m; the length is 750 m; the humidity is 75–85%; and the temperature is 20–23°C (Zhang et al., 2017).

According to the geological features and the historically tectonic movement nearby, the development periods of these two investigated caves were estimated as later than middle Pliocene (3.5–4 Mya), which fitted well with the estimation of the formation age of most caves in China (late Pliocene to early Pleistocene, ca. 3 Mya; Xiong and Zhu, 1994; Qin and He, 2004; Ping et al., 2011; Zhang and Zhu, 2012).

### Taxon Sampling, Molecular Data, and Phylogenetic Analysis

We included representative species of most lineages in Ascomycota with a total number of 167 species (**Supplementary Table S1**), including the 20 suspected obligate troglobitic fungi described by Zhang et al. (2017), and two species of Basidiomycota, *Boletus edulis* and *Rubroboletus sinicus*, as outgroup. Six loci were used for analysis, i.e., ITS, LSU, SSU, TEF, RPB1, and RPB2. Two new loci, SSU and RPB1, of these 20 species were amplified using primers NS1/NS4 (White et al., 1990) and RPB1-Cr/RPB1-Af (Matheny et al., 2002), respectively. Amplification reactions were performed according to the program of Zhang et al. (2017), except the annealing temperature, 54°C for SSU and RPB1.

All sequences of different loci were aligned using MAFFT^1^ (Katoh and Toh, 2010) and modified manually in MEGA v. 7 (Kumar et al., 2016) separately. Then, individual alignments were concatenated and used for phylogenetic analysis next step. Ambiguously aligned regions were excluded from all analyses.

Maximum likelihood (ML) and Bayesian inference (BI) methods were used to reveal the phylogenetic relationship. The ML analyses were executed using RAxML-HPC v. 8.2.7 (Stamatakis, 2014) with 1,000 replicates under the GTR-GAMMY model. The robustness of branches was assessed by bootstrap analysis with 1,000 replicates. For Bayesian analysis, the best model of evolution was estimated using jModelTest v. 2.1.7 (Guindon and Gascuel, 2003; Darriba et al., 2012). Posterior probabilities (PPs) (Rannala and Yang, 1996; Zhaxybayeva and Gogarten, 2002) were calculated by Markov chain Monte Carlo (MCMC) sampling in MrBayes v. 3.2.1 (Huelsenbeck and Ronquist, 2001), using the estimated evolutionary models. Six simultaneous Markov chains were run for 10,000,000 generations, and trees were sampled every 1,000th generations (totally resulting 10,000 trees). The first 2,000 trees, representing the burn-in phase of the analyses, were discarded and the remaining 8,000 trees were used to calculate PPs in the majority rule consensus tree. The final trees were visualized in TreeView (Page, 1996). All the sequences generated in this study were deposited in GenBank (**Supplementary Table S1**).

### Molecular Clock Analysis

We used five fossils with reliable age and accurate identification to estimate the divergence times. Each fossil age served as a minimum constraint. *Paleopyrenomycites devonicus* was discovered in thin-section preparations of lower Devonian [400 Mya (million years)] and considered to be the oldest ascomycete fossil (Taylor et al., 1999). Here it was used as the minimum divergence time of Pezizomycotina (Prieto and Wedin, 2013; Garnica et al., 2016). *Paleoophiocordyceps coccophagus* was described as the oldest fossil of animal parasitic fungi (99–105 Mya) with a striking morphological similarity to the asexual states of *Ophiocordyceps* (Sung et al., 2008). Fossils of *Calicium* and *Chaenotheca* were reported from Baltic amber dating back to 35–55 Mya (Rikkinen, 2003). The *Alectoria* fossil from the Baltic amber (35–40 Mya) was used as the calibration point for *Alectoria* clade (Magdefrau, 1957; De Paz et al., 2011; Prieto and Wedin, 2013).

Bayes MCMC algorithm was implemented to estimate divergence times using the data from multi-locus and according multiple fossil calibration nodes **Supplementary Datasheet S1**. The analysis was performed using BEAST v. 2.4.5 software package (Bouckaert et al., 2014). The tree topology was estimated by RAxML in the last step. We partitioned the data by gene using the general time reversible (GTR) substitution model for each partition, as estimated by jModelTest v. 2.1.7. We used the relaxed clock log normal model, specifying an exponential distribution for the ucld.mean parameter with a mean of 10.0 and offset 0. A birth-death tree prior was implemented (Divergence Time Estimation using BEAST^2^). All groups containing calibration points were regarded as monophyletic in beast analysis. Beast analyses were run for 50,000,000 generation; meanwhile, parameters and trees were logged every 1000 generations. Convergence, mixing, and effective sample sizes (ESS) of parameters were checked in Tracer v. 1.6.5 (Rambaut et al., 2014). Three repeat analyses were performed for accuracy and LogCombiner (Bouckaert et al., 2014) was used to combine the runs. The first 20% trees representing the starting and unreliable results were removed from the analysis and a maximum clade tree was created with TreeAnnotator v. 2.4.5 (Bouckaert et al., 2014). Age estimation is followed by their highest posterior density (HPD) in parentheses, which based on 95% confidence interval of all sample values.

Divergence time estimation calculates stem and node ages for each clade. Subsequently, the stem ages are considered as the time a group originated, or differentiated from its sister clade, and the node ages as a group started diversification.

## Results

### Phylogenetic Relationships

**FIGURE 2:**
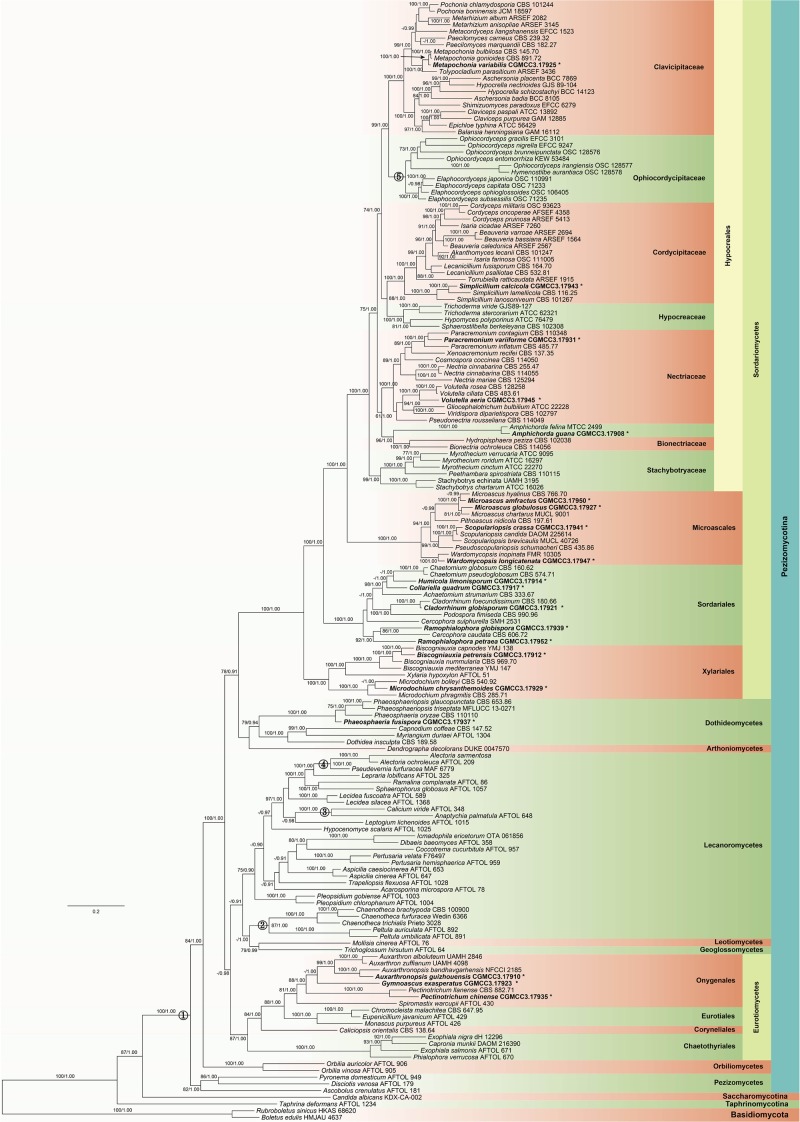
Maximum likelihood (ML) tree based on ITS, LSU, SSU, TEF, RPB1, and RPB2 sequences. ML bootstrap values (≥70%) and Bayesian posterior probability (≥90%) are indicated along branches (ML/PP). The tree is rooted with *Rubroboletus sinicus* and *Boletus edulis* (Basidiomycota). Numbers in the circles indicate the nodes used for fossil calibration: (1) *Paleopyrenomycites devonicus*; (2) *Chaenotheca* sp.; (3) *Calicium* sp.; (4) *Alectoria succinica*; and (5) *Paleoophiocordyceps coccophagus*. The suspected obligated fungal species are indicated in bold font and marked with “^∗^”. The major clades were marked with different colors and names are shown on the right side.

All of the major clades in Ascomycota were strongly supported with ML bootstrap proportion and Bayesian PPs (**Figure 2**) and the overall topology of the best tree generated in ML analysis is basically congruent with previous phylogenetic studies of Ascomycota (Hibbett et al., 2007; Sung et al., 2007; Schoch et al., 2009; Blackwell, 2010; Gazis et al., 2012; Prieto and Wedin, 2013). These 20 fungal species clustered into three classes and six orders which were well supported.

### Divergence Times

**FIGURE 3:**
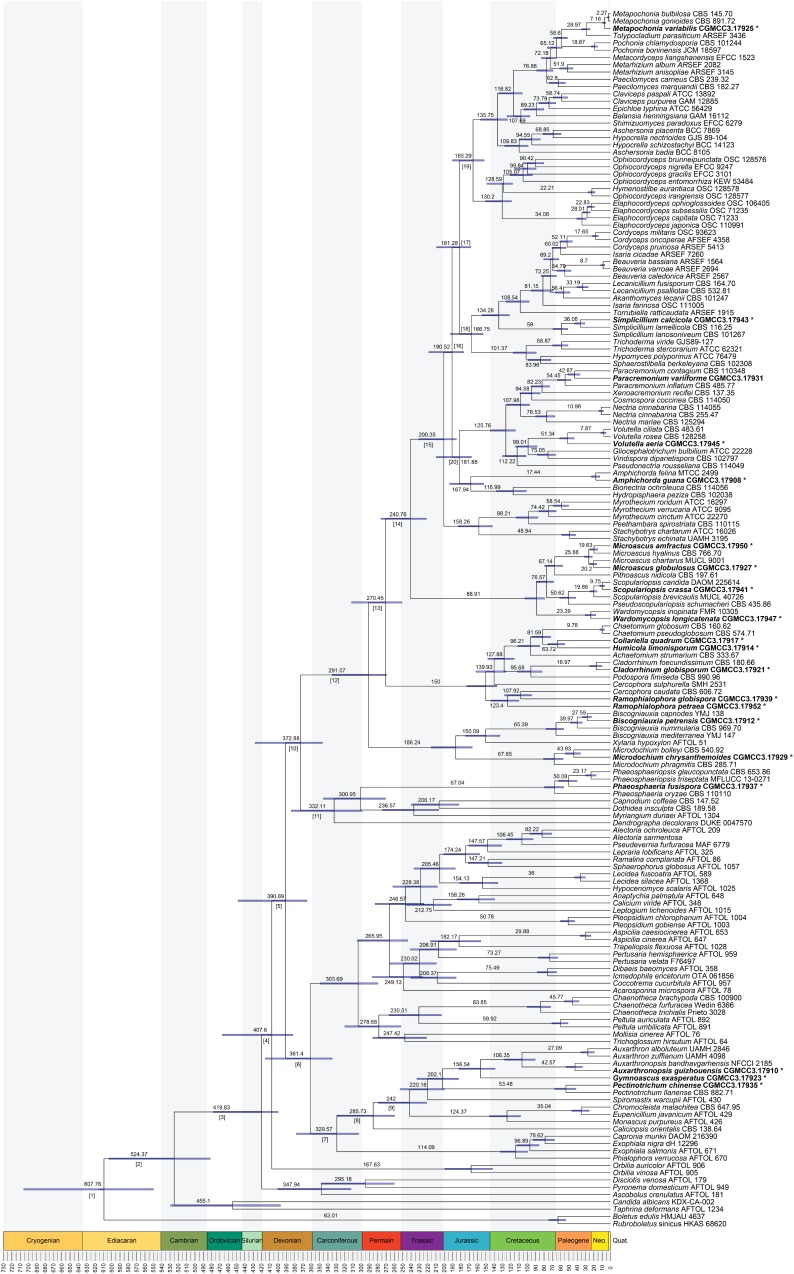
Maximum clade credibility chronogram of Ascomycota evolution. The chronogram is the result from the beast analysis based on the topology of ML tree. Each node represents the mean divergence time estimate (written above the nodes) and bars show the 95% HPD. Numbers corresponding to dated groups shown in **Table 1** are written above the nodes with square brackets. The suspected obligated fungal species are indicated in bold font and marked with “^∗^”.

The divergence of Basidiomycota and Ascomycota linage occurred in the Sinian to Early Cambrian (node 1; 608 Mya, 549–701.5 Mya for 95% HPD; **Figure 3** and **Table 1**). The first divergence in Ascomycota, i.e., the origin of Saccharomycotina, Taphrinomycotina, and Pezizomycotina, took place in Early Cambrian (node 2; 524 Mya, 481–601 Mya for 95% HPD). The earliest split in Pezizomycotina took place in Silurian (node 3; 420 Mya, 400–476 Mya for 95% HPD), resulting in Pezizomycetes and Pezizomycotina crown groups; in Silurian to Devonian (node 4; 408 Mya, 382–464 Mya for 95% HPD), resulting in Orbiliomycetes; in Carboniforous (node 6; 361 Mya, 334–413 Mya for 95% HPD), resulting in Eurotiomycetes and Lecanoromycetes; in Carboniferous (node 10; 373 Mya, 346–425 Mya for 95% HPD), resulting in Sordariomycetes crown (**Figure 3** and **Table 1**).

**Table 1:** Mean and range (95% HPD) divergence time estimations (Mya) of the major Ascomycota lineages.

Node label	Mean	95% HPD	Node label	Mean	95% HPD
1	607.76	548.91–701.46	11	332.11	297.24–382.13
2	524.37	481.31–600.56	12	291.07	268.62–332.4
3	419.83	400.00–476.09	13	270.45	249.23–308.75
4	407.6	381.57–464.21	14	240.76	221.41–275.18
5	390.89	364.87–445.37	15	200.35	184.91–229.36
6	361.4	333.77–412.68	16	190.52	175.80–217.62
7	329.57	302.47–376.62	17	181.28	166.71–206.89
8	285.73	259.31–327.66	18	166.75	152.34–191.03
9	242.00	220.81–278.22	19	165.29	151.72–189.06
10	372.88	346.03–424.85	20	181.88	166.44–207.94

Divergence times of the suspected obligate troglobitic fungi were different (**Figure 3** and **Table 2**). The most recent divergence is *Metapochonia variabilis*, occurred in Miocene (7.2 Mya, 5.3–9.2 Mya for 95% HPD), and the oldest is *Gymnoascus exasperates* in late Jurassic (157 Mya, 139–181 Mya for 95% HPD). While these two caves were developed within 4 Mya, clearly younger than the suspected obligate troglobitic fungi. In other words, the fungal speciation did not occurred in the caves, and cave fungi were most likely exogenetic. The estimated evolutionary rates of different loci and combined are detailed in **Table 3**.

**Table 2:** Mean and range (95% HPD) divergence time estimations (Mya) of the suspected obligate troglobitic fungi.

Node label	Strains No.	Source	Mean	95% HPD
*Amphichorda guana*	CGMCC3.17908^T^	Bat guano	17.44	12.76–22.96
*Auxarthronopsis guizhouensis*	CGMCC3.17910^T^	Air	42.57	33.11–53.19
*Biscogniauxia petrensis*	CGMCC3.17912^T^	Rock	27.59	22.63–33.73
*Cladorrhinum globisporum*	CGMCC3.17921^T^	Water	16.97	9.82–25.24
*Collariella quadrum*	CGMCC3.17917^T^	Soil	63.72	55.16–74.58
*Gymnoascus exasperates*	CGMCC3.17923^T^	Bat guano	156.54	139.07–181.04
*Humicola limonisporum*	CGMCC3.17914^T^	Soil	63.72	55.16–74.58
*Metapochonia variabilis*	CGMCC3.17925^T^	Soil	7.16	5.33–9.21
*Microascus amfractus*	CGMCC3.17950^T^	Plant debris	19.63	15.08–24.61
*Microascus globulosus*	CGMCC3.17927^T^	Bat guano	20.2	15.92–24.83
*Microdochium chrysanthemoides*	CGMCC3.17929^T^	Air	43.93	35.81–53.43
*Paracremonium variiforme*	CGMCC3.17931^T^	Water	42.87	36.23–50.75
*Pectinotrichum chinense*	CGMCC3.17935^T^	Soil	53.48	41.61–67.1
*Phaeosphaeria fusispora*	CGMCC3.17937^T^	Air	50.08	39.96–61.8
*Ramophialophora globispora*	CGMCC3.17939^T^	Plant debris	107.92	93.94–125.78
*Ramophialophora petraea*	CGMCC3.17952^T^	Rock	123.4	109.64–142.64
*Scopulariopsis crassa*	CGMCC3.17941^T^	Soil	9.75	7.07–12.75
*Simplicillium calcicola*	CGMCC3.17943^T^	Rock	36.06	30.29–42.77
*Volutella aeria*	CGMCC3.17945^T^	Air	51.34	43.73–61.07
*Wardomycopsis longicatenata*	CGMCC3.17947^T^	Air	23.39	17.28–30.31

*T indicates ex-type strains.*

**Table 3:** Range of estimated evolutionary rates with confidence intervals (site/Mya).

Gene	Mean	Min	Max	SD
Combined	0.0033	0.0025	0.0041	0.0005
ITS	0.0020	0.0016	0.0024	0.0003
LSU	0.0009	0.0007	0.0010	0.0001
RPB1	0.0034	0.0026	0.0041	0.0004
RPB2	0.0039	0.0029	0.0051	0.0006
SSU	0.0003	0.0002	0.00035	0.00003
TEF	0.0061	0.0035	0.0092	0.0016

## Discussion

### Hypothesis for the Origin of the Subterranean Fauna

The adaptation of organisms to caves or groundwater environments has been subject of many studies (Leys et al., 2003). Adaptive shift hypothesis and climatic relict hypothesis are two general hypotheses on the origin of the subterranean fauna. According to the climatic relict hypothesis, an epigean species preadapted to the underground life may survive in the subterranean refuge when the surface environment becomes unfavorable due to climate change. While under the adaptive shift hypothesis, a preadapted epigen species may actively enter the subterranean habitats to exploit new resources once they become accessible (Bellés, 1992; Leys et al., 2003; Juan and Emerson, 2010; Ribera et al., 2010). Leys et al. (2003) studied the evolution of subterranean diving beetles in Australia based on phylogeny and molecular clock method and found that the diving beetles were preadapted to the underground life, and when the drought occurred in the Early Pliocene, they survived and adapted to cave or groundwater life (climatic relict hypothesis). Ribera et al. (2010) investigated the origin and evolution of subterranean beetles of the tribe Leptodirini in Western Mediterranean based on mitochondrial and nuclear sequences. Their results suggested that the main lineages of Leptodirini had developed the necessary modifications for the hypogean life by the early-mild Oligocene. In another word, they had preadapted to the underground life. In contrast to most current assumptions on the evolution of the underground fauna, there was no evidence for the extinction of epigean populations (adaptive shift hypothesis). Johnson et al. (2011) reported a new eel-like fish, *Protanguilla palau*, from an undersea cave in Palau and divergence time estimation showed that *Protanguilla* lineage diverged about 200 Mya, much earlier than the formation age of Palau-Kyushu Ridge (60–70 Mya). Accordingly, it has been suggested that the *P. palau* possibly had a considerably broader distribution than previous assumption.

Similar to the cave fauna, to determine the origin of cave fungi, it is important to determine if the true troglobitic fungi exist. Currently, there are 36 suspected troglobitic fungi reported, including the troglobitic fungi summarized by Vanderwolf et al. (2013) and the new cave fungi published by Zhang et al. (2017). Although these species might be discovered from non-cave environment in subsequent studies, they do stand as the currently most reasonable suspect of troglobitic fungi. *Geomyces destructans*, the most famous “cave fungus” previously, was isolated from bats outside caves by Dobony in 2012 (Vanderwolf et al., 2013). Comparing the divergence time of suspected troglobitic fungi and cave development geologic age is one of the best ways to infer the origin of cave fungi.

### Origin of Cave Fungi

The divergence time of Ascomycota and Basidiomycota estimated in our analysis (**Figure 3**) is similar to that inferred from previous analyses (Berbee and Taylor, 2001; Dhanasekaran et al., 2006; Taylor and Berbee, 2006; Floudas et al., 2012; Prieto and Wedin, 2013; Garnica et al., 2016). Divergence of our 20 suspected troglobitic fungi was estimated to be no later than Miocene (7.2 Mya), which is much earlier than cave development age (3.5–4.0 Mya). The speciation of these cave fungi was thus unlikely to occur in caves. This provided reasonably support for the speculation of Zhang et al. (2017) that cave fungi may have an origin of external environment.

Considering the features of subterranean habitat and outside environment, allopatric speciation appears to be a reasonable explanation for cave fauna (Mayr, 1966; Barr and Holsinger, 1985; Giraud et al., 2008). This, however, cannot explain cave fungi. The geographic age of caves is obviously too short for a most fungal speciation processes. Apparently, the constantly lower temperature (Rohde, 1992; Gillman and Wright, 2006; Gillman et al., 2009; Goldie et al., 2010; Wright et al., 2010) and extremely limited energy resources also lead to a limited microevolution in caves (Goldie et al., 2010). Unfortunately, nowadays, studies of the evolution speed were mainly on plants and animals.

The data obtained from this study well demonstrated the non-cave origin of cave fungi, as the caves were generally believed to be formed later than Pliocene period (3.5–4.0 ma), which is shown to be much shorter than the most recently speciated species *M. variabilis* (late Miocene, ca. 7.2 Mya). It is therefore concluded that the new species described from cave are unlikely troglobitic fungi but travelers from other environments, although they have not been reported from a terrestrial environment.

Although some new species have been discovered from caves, hitherto no new genus was described from caves. Even within the described new species, none of the any two were shown to be sister lineages (Zhang et al., 2017), indicating no evidence of fungal evolutionary divergence in caves. Within the currently descried new species from caves, we could not recognize any distinct morphological and physiological feature that might be potentially troglobitic. Future studies employing metagenomics techniques could possibly bring broader and more conclusive data to answer above questions.

## Author Contributions

Z-FZ designed the work that led to the submission, analyzed the data, and drafted most part of the manuscript. PZ revised our manuscript. LC conceived the work, drafted part of the manuscript, and revised our manuscript.

## Conflict of Interest Statement

The authors declare that the research was conducted in the absence of any commercial or financial relationships that could be construed as a potential conflict of interest.

## Supplementary Material

TABLE S1 Specimens used in this study with GenBank accession numbers (xlsx).

DATASHEET S1 Information about the priors and parameters used in BEAST to obtain the divergence time.
